# Ginsenoside Rb2 improves insulin resistance by inhibiting adipocyte pyroptosis

**DOI:** 10.1080/21623945.2020.1778826

**Published:** 2020-06-24

**Authors:** Yi Lin, Yepeng Hu, Xiang Hu, Lijuan Yang, Xueqin Chen, Qianqian Li, Xuejiang Gu

**Affiliations:** Department of Endocrine and Metabolic Diseases, 1st Affiliated Hospital of Wenzhou Medical University, Wenzhou City, Zhejiang Province, P.R. China

**Keywords:** Ginsenoside Rb2, insulin resistant, pyroptosis, NLRP3 inflammasome, NF-Κb signalling pathway

## Abstract

Pyroptosis plays a critical role in the development of obesity-associated inflammation and insulin resistantance (IR). Ginsenoside Rb2 (Rb2), the main component of ginsenosides has drawn appreciable interest in the context of glucose metabolism. In the present study, we investigated Rb2-mediated protection against obesity-induced IR and the related mechanisms. Rb2 could significantly reduce high-fat diet (HFD)-induced body weight changes, fat accumulation and IR. In addition, Rb2 treatment inhibited pyroptosis-related genes and proteins, such as caspase-1, ASC, NLRP3, IL-1β and GSDMD in HFD-fed mice. The above results were recapitulated in 3T3-L1 adipocytes and demonstrated that Rb2 improved TNF-α induced IR and pyroptosis in 3T3-L1 adipocytes. Furthermore, Rb2 reduced the phosphorylation levels of p65 and IκBα both in vitro and in vivo. The present study showed that Rb2, which could serve as a promising agent for the treatment of IR and obesity, ameliorated IR by inhibiting pyroptosis in adipocytes in vivo and in vitro through the NF-κB pathway.

## Introduction

Obesity, which is caused by the accumulation of white adipose tissue (WAT), leads to numerous metabolic disorders, such as diabetes mellitus, fatty liver disease, and cardiovascular disease and thus has become a public health concern [1,2]. Importantly, the growing evidence obtained over the last few years has unambiguously shown that adipose tissue, especially visceral adipose tissue, is the point of insulin resistance (IR) [3–5]. In obesity, adipocytes may become hypertrophy, hypoxia or death, which can result in severe dysfunction of adipose tissue (AT) and impairment of insulin sensitivity [2,6,7].

A previous study noted that the NOD-like receptor pyrin containing 3 (NLRP3) inflammasome is a culprit of obesity and IR and that obese Nlrp3-/- knockout (KO) mice are more insulin sensitive than obese wild-type mice [8]. The NLRP3 inflammasome, in most cases, escalates the intensity of sterile inflammatory responses and leads to the inflammatory form of programmed cell death originally termed ‘pyroptosis’ [9]. Pyroptosis is distinguished by cellular lysis, and pore formation in the plasma membrane, which results in release of proinflammatory and pyrogenic cytokines, including interleukin-1β (IL-1β) and IL-18, to mediate robust immune response [10]. In addition, there is a report that proposed uncontrolled adipose tissue inﬂammation and IR may be caused by pyroptosis in hypertrophic adipocytes and the release of proinﬂammatory cytokines during nutritional surplus [11].

As one of the main components of ginsenosides, ginsenoside Rb2 (Rb2) has many beneficial biological effects such as anti-apoptosis, anti-inflammation, and anti-cancer [12–14]. Especially, Rb2 has been studied in glucose and lipid metabolism for a long time [15,16]. Our recent study revealed that Rb2 could attenuate insulin resistance both in vitro and vivo via phosphorylation of AKT and inhibited NF-κB signalling pathway [17]. The other article of our group showed that Rb2 improved insulin sensitivity by activation of brown fat and induction of browning of white Fat [18]. However, there are few researches on exploring the mechanism that Rb2 improves IR on pyroptosis.

Thus, we designed the present study to investigate the effects of Rb2 on IR and pyroptosis in adipocytes and explore the potential molecular mechanisms to provide new therapeutic targets for the treatment of obesity and IR.

## Materials and methods

### Materials

Rb2 (purity >98.0%) was obtained from Shanghai Yuanye Biotech Co. Ltd. (Shanghai, MO, China). Foetal bovine serum (FBS) and Dulbecco’s modified Eagle’s medium (DMEM) were obtained from Gibco Laboratory (Gaithersburg, MD), while dexamethasone and isobutylmethylxanthine (IBMX) were purchased from Sigma (St. Louis, MO, USA). Recombinant murine TNF-α was obtained from PeproTech(New Jersey, USA). Antibodies including rabbit anti-GAPDH (#2128), anti-apoptosis-associated speck-like protein (ASC) (#67,824), anti-IκBα (#AF5002) and anti-phospho-IκBα (#AF2002) antibodies were purchased from Affinity (Danvers, MA, USA). Antibodies against NLRP3 (#ab210491) and GSDMD (#ab209845) were obtained from Abcam (Cambridge, UK). A mouse anti-caspase-1 antibody (sc-514) was purchased from Santa Cruz Biotechnology (Dallas, TX, USA). The following items were purchased from Cell Signalling Technology (Danvers, MA, USA): an anti-AKT antibody (#4691) and an anti-phospho-AKT (Ser473) antibody (#4060).

### Animals

Six-week-old male C57BL/6 J mice were purchased from the Shanghai Slake Experimental Animal Co. Ltd. and kept under comfortable conditions with a 12-h light/dark cycle. Mice were allowed access to water and a chow diet consisting of 10% fat (SLAC, Shanghai) or a HFD (60% of Kcal from fat, 12492i, USA) for 10 weeks. Based on the above situations, mice were assigned into three groups: mice fed the standard rodent chow diet (CD) and intraperitoneally injected with phosphate-buffered saline (PBS) were defined as the normal chow group, obese mice fed the HFD and intraperitoneally injected with vehicle (PBS) were defined as the HFD group (HFD+PBS), and obese mice fed the HFD and intraperitoneally injected with 40 mg/kg/d Rb2 for 10 days were defined as the drug treatment group (HFD + Rb2). At the end of experiments, body weight, glucose tolerance and insulin tolerance were measured. This study was approved by the Institutional Animal Care and Use Committee at Wenzhou Medical University.

### Cell culture and differentiation

The mouse 3T3-L1 preadipocyte line was obtained from the American Type Culture Collection (Rockville, MD) and cultured in DMEM supplemented with 10% FBS, penicillin and streptomycin at 37°C. For cell differentiation, two days after reaching confluence, cells were cultured with differentiation medium (DMEM supplemented with 10% FBS, 1 µM dexamethasone, 0.5 mM IBMX and 10 μg/ml insulin). Then, the cells were incubated with an induction medium containing 10 µg/mL insulin and 10% FBS.

### Glucose tolerance test (GTT) and insulin tolerance test (ITT)

For the GTT, mice were fasted for 12 h. Then, the mice were injected intraperitoneally with glucose (0.75 g/kg), and their blood glucose level at 0, 15, 30, 60, and 120 min was detected by tail bleeding. For the ITT, mice were fasted for 2 h. Then, the mice were injected intraperitoneally with insulin (HumulinR, 0.75 U/kg), and their blood glucose level at 0, 15, 30, 60, and 120 min was detected by tail bleeding.

### RNA isolation and quantitative RT-PCR

Total RNA was extracted with Trizol (Invitrogen, Carlsbad, CA, USA) from adipose tissue samples and cells and then reverse transcribed into cDNA using the RevertAid First Strand cDNA Synthesis Kit (Thermo, USA). Quantitative real-time assays were carried out using the synthesized cDNA and the CFX96 Real-Time PCR Detection System (Bio-Rad, CA, USA). The primers for the genes were as shown in Table 1:

**Table 1.** Primer sequences used in the qPCR experiments

Gene name	Primer sequences (5ʹ-3ʹ)
GAPDH	Forward ATCATCTCCGCCCCTTCTGC Reverse ATGCCTGCTTCACCACCTTC
Caspase1	Forward ACAAGGCACGGGACCTATG Reverse TCCCAGTCAGTCCTGGAAATG
NLRP3	Forward GAGCTGGACCTCAGTGACAATGC Reverse ACCAATGCGAGATCCTGACAACAC
ASC	Forward GAAGTGGACGGAGTGCTGGATG Reverse CTTGTCTTGGCTGGTGGTCTCTG
GSDMD	Forward ACTGAGGTCCACAGCCAAGAGG Reverse GCCACTCGGAATGCCAGGATG
IL-1β	Forward TCGCAGCAGCACATCAACAAGAG Reverse TGCTCATGTCCTCATCCTGGAAGG
Adiponectin	Forward CCAATGTACCCATTCGCTTTAC Reverse GAAGTAGTAGAGTCCCGGAATG
Glut4	Forward TATTCAACCAGCATCTTCGAGT Reverse GTCCAGCTCGTTCTACTAAGAG
IRS-1	Forward GAGTTGAGTTGGGCAGAATAGG Reverse CCTATCTGCATGGTCATGTAGT

### Protein extraction and western blot analysis

Total protein was extracted from adipose tissue samples and cells with RIPA lysis buffer and quantified by a BCA assay kit (Thermo, USA). For western blotting, protein samples of equal amounts (30 μg) were separated by SDS-PAGE, transferred to PVDF membranes (Millipore Corporation, MA, USA), blocked with 5% skim milk for 2 h at room temperature, and incubated with different primary antibodies at 4°C overnight. Subsequently, the membranes were incubated with an anti-rabbit/mouse IgG horseradish peroxidase-conjugated secondary antibody for 1 h at room temperature. Finally, the membranes were detected using an ECL detection reagent (Thermo Scientific Pierce, USA) and analysed by Image Lab software (Bio-Rad, Hercules, CA, USA).

### Histology and immunohistochemistry

Fresh adipose tissue samples from each mouse were fixed in 4% paraformaldehyde, embedded in paraffin, and sectioned into 4-μm-thick sections. After incubating with 3% hydrogen peroxide (H_2_O_2_) for 10 min and blocking with goat serum, the sections were stained with an anti-ASC antibody (1:200) overnight at 4°C and exposed to biotinylated secondary antibodies for 30 min at room temperature. Finally, the tissue sections were incubated with diaminobenzidine (DAB), counterstained with haematoxylin and imaged with a light microscope (Olympus BX53, Tokyo, Japan).

### Cell viability assay

3T3-L1 cells were seeded at a density of 1 × 10^3^ cells/well in triplicate in 96-well plates. After the 3T3-L1 preadipocytes were induced to differentiate into mature adipocytes, the cells were treated with TNF-α (5 ng/mL) and different concentrations of Rb2 (0, 10, 25, 50 or 100 μM). Cell viability was examined by using a CCK-8 kit (Toyobo, Japan) according to the manufacturer’s instructions. Briefly, 10 μl of CCK-8 reagent was added to each well and then incubated for 1 h in the dark. The absorbance at 450 nm was measured with a microplate reader (SpectraMax Plus384, Molecular Devices, USA).

### Statistical analysis

The data were expressed as the mean±SEM. The differences among groups were analysed by one-way ANOVA using GraphPad Prism 5 (San Diego, CA, USA). P < 0.05 was considered to be statistically significant.

## Results

### Rb2 alleviated body weight and improved the insulin sensitivity of WAT in HFD-fed mice

**Figure 1.**
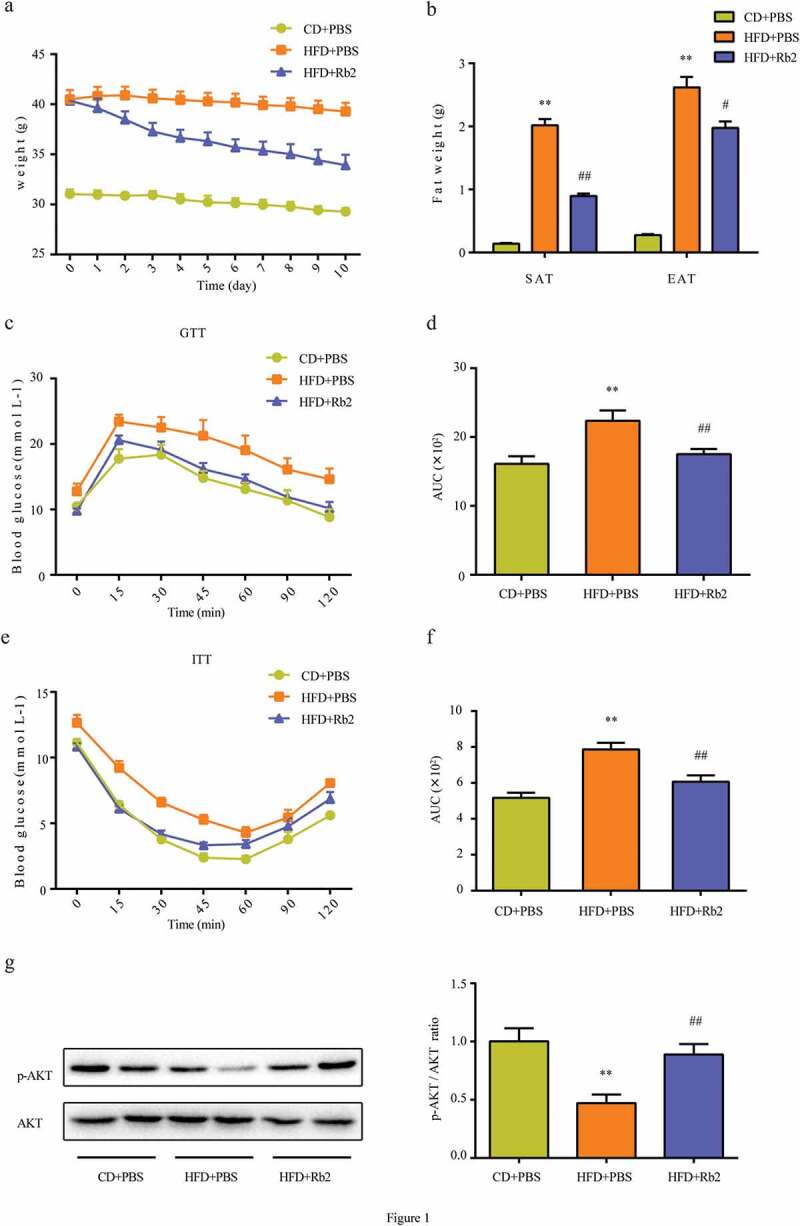
Rb2 attenuated diet-induced obesity in C57BL/6 J mice. (a) Body weights of control mice and HFD-fed mice treated with or without Rb2 (40 mg/kg/d) for 10 days. (b) Weights of SAT and EAT after 10-day treatment. Performance of GTT (c), and ITT (e). Area under curve (AUC) of GTT (d) and ITT (f). (g) Phosphorylation (p-AKT) and total AKT levels in epididymal adipose tissue (EATs), ratio of phospho-AKT/total AKT. N = 5 per group. Data are presented as mean ± SEM, *P < 0.05, **P < 0.01 compared with CD+PBS group; ^#^P < 0.05, ^##^P < 0.01 compared with HFD+PBS group

To explore the therapeutic efficacy of Rb2, we fed mice a HFD to establish an obesity model. HFD-induced body weight gain was obviously alleviated after 10 days of 40 mg/kg Rb2 treatment (Figure 1(a)). In agreement with the body weight results, body fat mass was greatly increased in HFD-fed mice, and this increase tended to be reversed by Rb2 treatment (Figure 1(b)). In addition, we demonstrated that the HFD induced adipocytes to become hypertrophic in the adipose tissue, while Rb2 effectively ameliorated adipocyte size and morphology (Figure 1s). To understand how Rb2 improved glucose tolerance, we performed an ITT and a GTT. The results revealed that Rb2 improved glucose tolerance (Figure 1(c-d)) and insulin sensitivity (Figure 1(e-f)) in HFD-fed mice. Additionally, an analysis of Akt expression in epididymal adipose tissue (EAT) showed that Rb2 intervention reversed the HFD-induced reduction in AKT phosphorylation levels (Figure 1(g))

### Rb2 displayed an antipyroptotic ability in EAT

**Figure 2.**
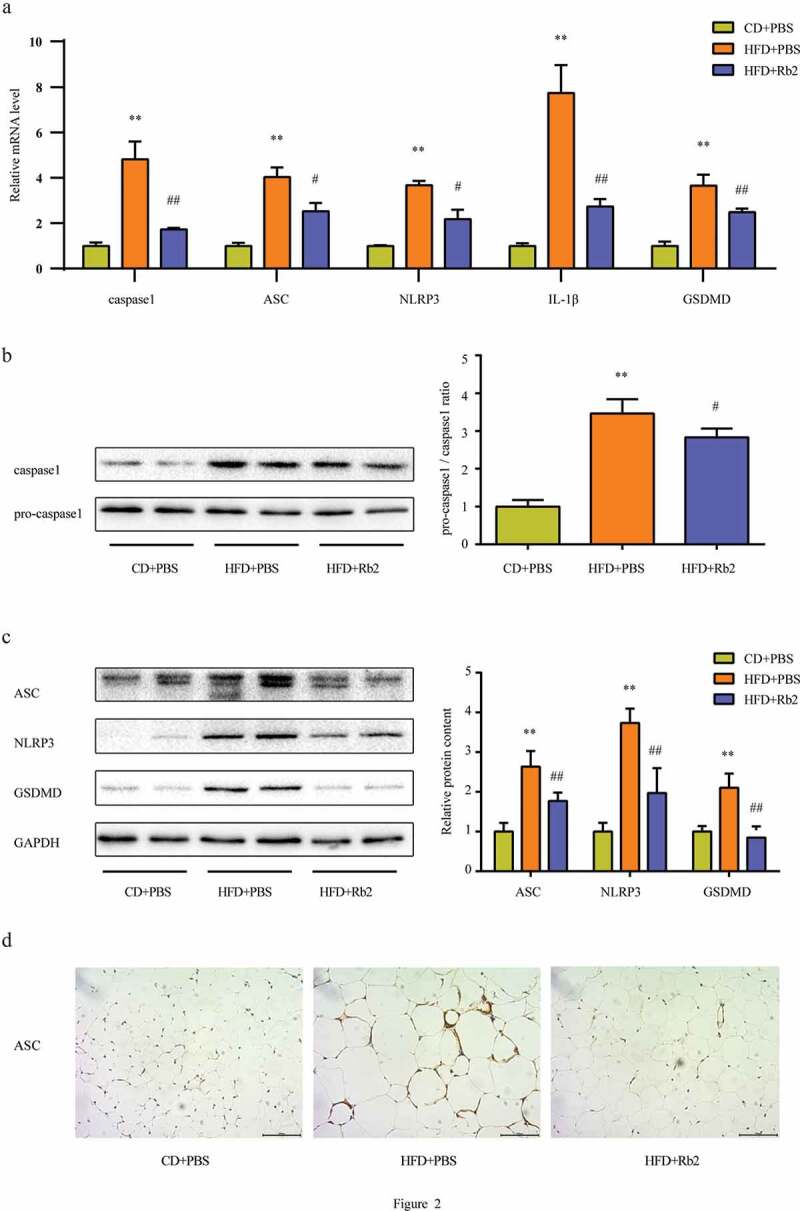
Rb2 inhibited adipocyte pyroptosis in epididymal adipose tissue (EATs). (a) Increased mRNA expression levels of caspase 1, ASC, NLRP3, IL-1β and GSDMD in HFD-fed mice EAT, which can be ameliorated by Rb2. (b) Pro-caspase 1 and total caspase 1 levels, ratio of pro-caspase 1/caspase 1. (c) ASC, NLRP3 and GSDMD levels. (d) ASC staining from EAT secetion. N = 5 per group. Data are presented as mean ± SEM. *P < 0.05, **P < 0.01 compared with CD+PBS group; ^#^P < 0.05, ^##^P < 0.01 compared with HFD+PBS group. GSDMD is a quintessential mediator of pyroptosis

It is well known that the activation of the NLRP3 inflammasome is essential for triggering pyroptosis and GSDMD is a quintessential mediator of pyroptosis; therefore, we examined the mRNA levels of caspase-1, ASC, NLRP3, GSDMD and interleukin (IL)-1β. HFD-fed mice exhibited markedly increased cleaved caspase-1, NLRP3, ASC and GSDMD levels in EAT, and the administration of Rb2 ameliorated these increases (Figure 2(a)). Consistent with the mRNA expression results, compared with the HFD group, the Rb2-treated group showed suppressed expression of related proteins (Figure 2(b-c)). The alteration in ASC protein expression was also supported by the results of immunohistochemistry staining of the EAT (Figure 2(d)). Since growing evidence indicates that the NLRP3 inflammasome is a culprit in IR, Rb2 may improve IR by inhibiting pyroptosis in EAT.

### Rb2 restored the viability of 3T3-L1 adipocytes exposed to TNF-α in vitro

**Figure 3.**
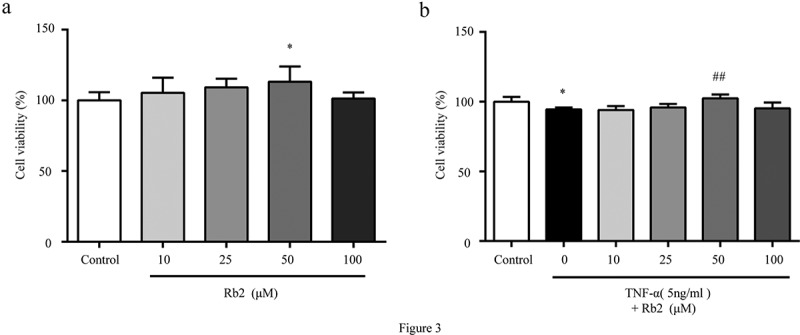
Rb2 promoted growth in 3T3-L1 adipocytes. Cell viability were measured by Cell Counting Kit-8 (CCK-8) in 3T3-L1 adipocytes with various Rb2 concentrations (10, 25, 50 or 100 μM) in the absence (a) or presence of TNF-α (5 ng/mL) (b). Data are presented as mean ± SEM. *P < 0.05, **P < 0.01 compared with control group; ^#^P < 0.05, ^##^P < 0.01 compared with TNF-α group

Cell viability experiments showed that Rb2 treatment increased cell viability in a dose-dependent manner. As shown in Figure 3(a), the cell viability of the 50 μM Rb2 group was significantly higher than that of the control group and other Rb2 treatment groups. However, Rb2 exhibited cytotoxicity when the concentration was raised to 100 μM. Furthermore, a CCK8 assay was performed with TNF-α-induced insulin-resistant adipocytes (Figure 3(b)). The results demonstrated that TNF-α obviously inhibited cell viability, while Rb2 treatment increased cell viability in a dose-dependent manner, but 100 μM Rb2 inhibited cell viability, suggesting that a high concentration (100 μM) of Rb2 could aggravate TNF-α-induced injury in 3T3-L1 cells by increasing cytotoxicity. Therefore, we treated cells with 50 μM Rb2 in subsequent experiments.

### Rb2 improved 3T3-L1 adipocytes insulin sensitivity

**Figure 4.**
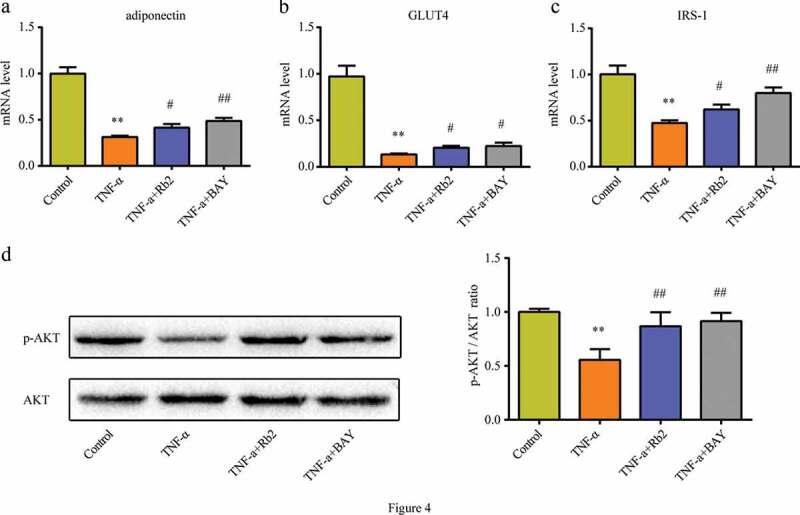
Rb2 improved insulin sensitivity in 3T3-L1 adipocytes. mRNA levels of adiponectin(a), GLUT4 (b) and IRS-1(c) in 3T3-L1 adipocytes. (d) Phosphorylation (p-AKT) and total AKT levels, p-AKT/AKT ratio in 3T3-L1 adipocytes. Data are presented as mean ± SEM. *P < 0.05, **P < 0.01 compared with control group; ^#^P < 0.05, ^##^P < 0.01 compared with TNF-α group. BAY 11–7082 (BAY) is an NF-κB pathway inhibitor

To further determine the effects of Rb2 on the IR of adipocytes, 3T3-L1 adipocytes were pretreated with Rb2 or control medium for 1 hour and then stimulated with TNF-α for 3 days. This study showed that Rb2 treatment ameliorated TNF-α-induced impairments in insulin signalling genes, such as adiponectin, GLUT4 and IRS-1 (Figure 4(a-c)). Consistently, adipocytes subjected to TNF-α had lower expression of p-Akt than unstimulated adipocytes, but Rb2 treatment attenuated this alteration (Figure 4(d)).

### Rb2 displayed an antipyroptotic ability in adipocytes

**Figure 5.**
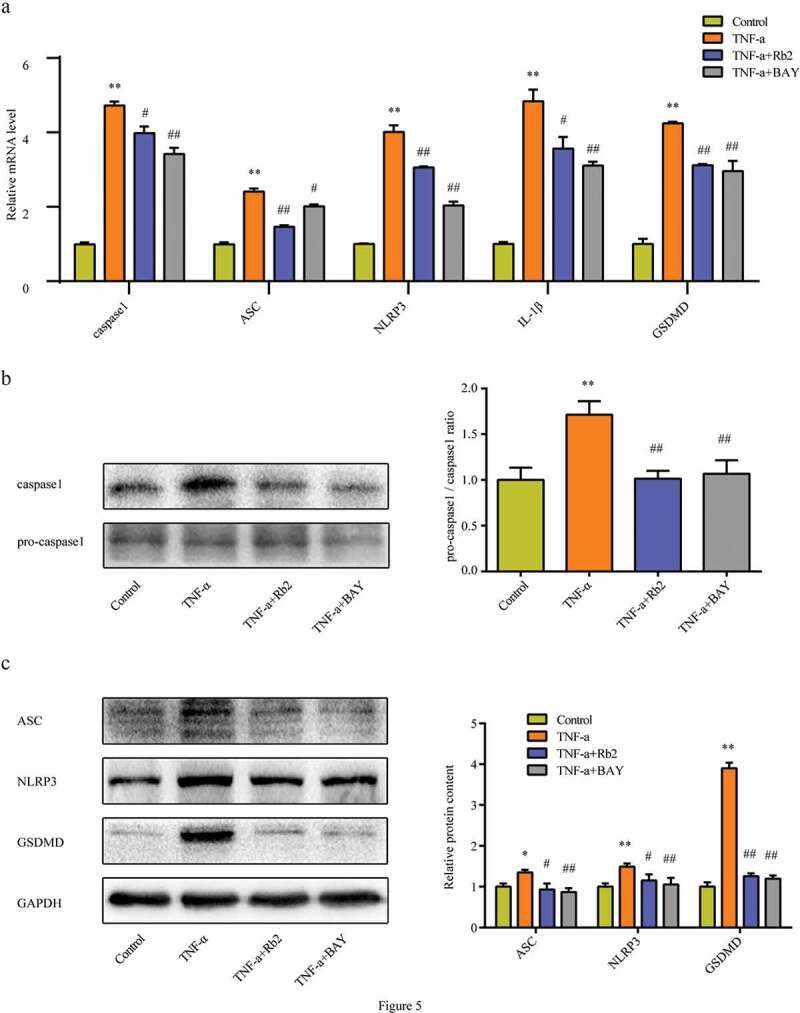
Rb2 inhibited pyroptosis in 3T3-L1 adipocytes. (a) mRNA levels of caspase 1, ASC, NLRP3, IL-1β and GSDMD. (b) Pro-caspase 1 and caspase 1, ratio of pro-caspase 1 to caspase 1. (c) ASC, NLRP3 and GSDMD levels in 3T3-L1 adipocytes. Data are presented as mean ± SEM. *P < 0.05, **P < 0.01 compared with control group; ^#^P < 0.05, ^##^P < 0.01 compared with TNF-α group. GSDMD is a quintessential mediator of pyroptosis

As shown in Figure 5(a), TNF-α treatment stimulated the mRNA expression of caspase-1, ASC, NLRP3, GSDMD and IL-1β. In addition, Rb2 treatment could significantly suppress this production induced by TNF-α. Consistent with the mRNA expression results, Rb2 treatment inhibited the protein expression of these molecules, as detected by western blot analysis (Figure 5(b-c)). Combining the above results, it could be shown that the protective effects of Rb2 on IR were related to the inhibition of pyroptosis in adipocytes.

### Rb2 inhibited pyroptosis via the NF-κB pathway

**Figure 6.**
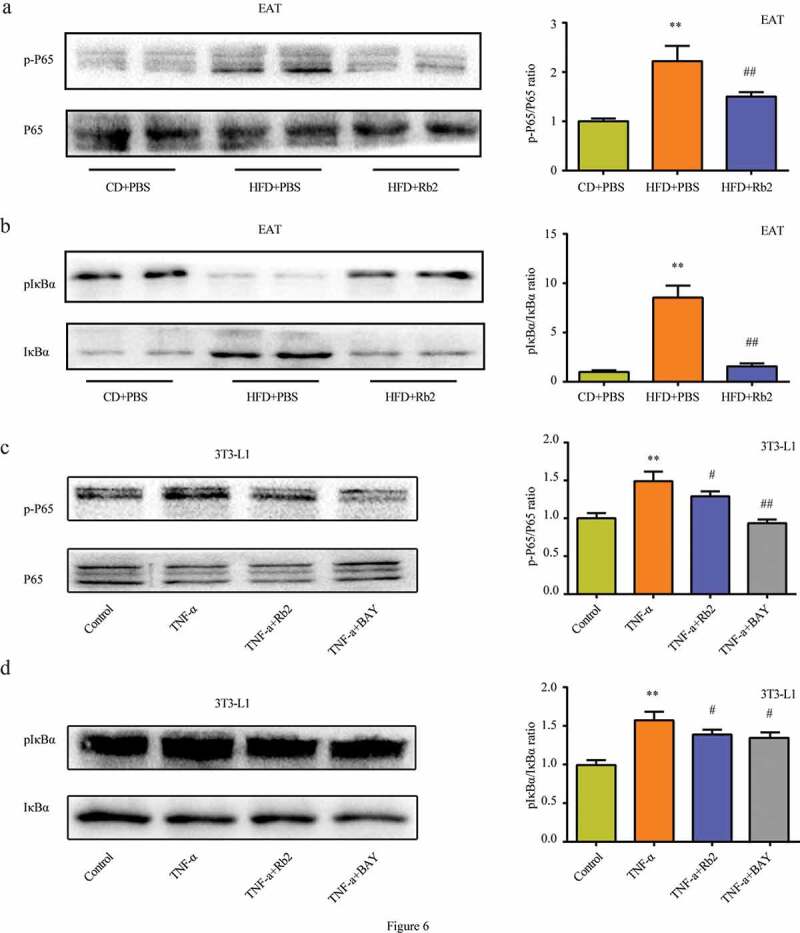
Rb2 inhibited NF-κB pathway both in epididymal adipose tissue (EATs) and 3T3-L1 adipocytes. Phosphorylation (p-P65) and total NF-κB/P65 (P65) levels in EATs (a) and 3T3-L1 cells (c); Phosphorylatio (p-IκBα) and total IκBα levels in EATs (b) and 3T3-L1 cells (d). Data are presented as mean ± SEM. (a-b) *P < 0.05, **P < 0.01 compared with CD+PBS group; ^#^P < 0.05, ^##^P < 0.01 compared with HFD+PBS group; (c-d)*P < 0.05, **P < 0.01 compared with control group; ^#^P < 0.05, ^##^P < 0.01 compared with TNF-α group

The NF-κB signalling pathway is a crucial regulator of NLRP3 inflammasome formation. The study showed that Rb2 treatment could effectively reduce the HFD-induced phosphorylation of p65 and IκBα in vivo (Figure 6(a-b)) and that the phosphorylation levels of p65 and IκBα also decreased in the presence of Rb2 in vitro (Figure 6(c-d)). In addition, BAY 11–7082 (BAY), an NF-κB pathway inhibitor, was shown to improve IR (Figure 4(a-d)) and inhibit pyroptosis in adipocytes (Figure 5(a-c)). These data indicated that inhibiting the NF-κB pathway played an essential role in the suppression of pyroptosis by Rb2 treatment

## Discussion

Rb2, which possesses various bioactivities including anti-inflammatory, anti-cancer and anti-obesity activities, has been studied for a long time [13,15,18,19]. However, there is little research exploring the mechanism by which Rb2 improves IR in obese mice. The present study demonstrated that Rb2 significantly ameliorated IR in HFD-fed mice by reducing cell pyroptosis. This beneficial role of Rb2 was further supported by in vitro results, which demonstrated that Rb2 exerted potential protective activities in 3T3-L1 cells exposed to TNF-α by reducing cell pyroptosis through regulating the NF-κb pathway. Thus, our data showed that Rb2 is involved in regulating pyroptosis of adipocytes upon the obesity state to ameliorate IR.

Obesity, including HFD-induced obesity, is considered a risk factor for numerous chronic diseases, with the most common diseases being diabetes, cardiovascular disease and metabolic syndrome [20,21]. With chronic overnutrition, excessive fatty nutrition induces adipocytes to become hypertrophic [22,23]. In the present study, we demonstrated that HFD-induced adipocytes became hypertrophic in adipose tissue, while Rb2 efficaciously ameliorated adipocyte size and morphology changes. As overfeeding processes escalate, hypertrophic adipocytes eventually overfill, yielding necrotic cellular debris and large lipid droplets to the detriment of neighbouring tissues, which play important roles in impairing insulin sensitivity. Indeed, the present report showed that Rb2 was effective in the control of weight gain and blood glucose levels in HFD-fed mice, which corroborated our previous study [17,18]. To further observe the effects of Rb2 on IR in vitro, we treated 3T3-L1 adipocytes with a TNF-α intervention to induce a classic IR cell model. The results found that TNF-α inhibited the expression levels of insulin sensitivity-related genes, such as adiponectin, GLUT4 and IRS-1, while Rb2 treatment remarkably increased the expression of these genes. Insulin can promote Akt phosphorylation to regulate glucose uptake [24]. Interestingly, our results also revealed that TNF-α significantly downregulated the expression of p-AKT, while Rb2 treatment enhanced p-AKT expression. These results indicate that Rb2 treatment directly ameliorates IR.

In subsequent experiments, we explored the mechanism by which Rb2 inhibits IR. Pyroptosis is a newly identified form of cell death characterized by cell swelling, plasma membrane disruption, and massive proinflammatory cytokine leakage [10,25]. Pyroptosis has been observed not only in monocytes and dendritic cells infected with pathogenic microorganisms [26] but also in nonmacrophage cells after non-infectious stimulation [27,28]. Recently, it was reported that adipose tissue also undergoes pyroptosis [11,29]. In addition, Hersoug et al. proposed that adipocyte death size is defined by the intracellular concentration of LPS that triggers pyroptosis [30]. This proposal is in agreement with a previous report that showed that white adipocyte overexpansion induces a stress state that ultimately leads to obese adipocyte death by pyroptosis, which recruits macrophages to the adipose tissue and induces inflammation and IR in obese mice [11]. Pyroptosis requires the proteolytic activation of caspase 1. Caspase 1 activation can release IL-1β) and IL-18 by the rapid loss of cell membrane integrity, subsequently inducing cell pyroptosis. Concordantly, the present study showed that Rb2 treatment significantly inhibited both HFD-induced and TNF-α-induced activation of caspase1 in vivo and in vitro.

Generally, caspase 1 activation requires the NLRP3 inflammasome. The NLRP3 inflammasome, which is significantly related to pyroptosis, contains inactive NLRP3, ASC and procaspase 1. Accordingly, several studies have reported associations between the NLRP3 inflammasome and obesity, IR and type 2 diabetes mellitus (T2DM) [31,32]. In agreement with this information, most studies indicate that NLRP3 expression is increased in the adipose tissue of HFD-fed mice, while a calorie-restricted diet seems to decrease the expression of this gene [8]. To evaluate the impacts of Rb2 on inflammasome pathway of adipocytes, we measured the NLRP3 inflammation genes expression in adipose tissue. We found that feeding mice a HFD increased the mRNA and protein expression levels of the components of the NLRP3 inflammasome, including NLRP3 and ASC, in EAT. Treatment with Rb2 significantly inhibited the HFD-induced levels of these genes in EAT. Taken together, the growing body of evidence indicates that Gasdermin-d (GSDMD), an essential candidate for pore formation that causes cell swelling and eventual lysis, is a quintessential mediator of pyroptosis [33–35]. Notably, the present study showed that Rb2 administration reduced HFD-induced GSDMD expression augmentation.

Adipose tissue is a heterogeneous tissue, which is composed of adipocytes and other cell types, such as adipose tissue macrophages (ATMs) [36,37]. However, it is not known whether the activation of the NLRP3 inflammasome occurs in adipocytes or ATMs in adipose tissue. Many articles have focused on the expression of inflammasome components in ATMs [38,39], while other studies have proven that inflammasome activation occurs in adipocytes [29]. Therefore, we detected the expression of ASC, an important component of the NLRP3 inflammasome, in adipose tissue by immunohistochemistry. The results showed that the ASC protein was expressed in both adipocytes and ATMs in HFD-fed mice, while Rb2 treatment reduced this expression. These findings were in accordance with those of another study in which the activation of NLRP3 inflammasomes occurred in both adipocytes and ATMs, but the adipocyte inflammasomes activated earlier, which promoted obesity-induced IR [40]. Therefore, we focused on the activation of adipocyte inflammasomes and found that TNF-α treatment induced adipocyte pyroptosis while also inducing IR in vitro, indicating that pyroptosis in adipocytes plays a key role in the development of IR. Furthermore, we observed that Rb2 treatment could decrease TNF-induced pyroptosis. Thus, our data indicated that adipocyte pyroptosis was central to Rb2 performing its anti-IR function.

We further explored the potential molecular mechanism underlying the regulation of pyroptosis by Rb2 treatment. Accumulating studies have demonstrated that NF-κB signalling is the critical sensor for upregulating the transcription of NLRP3 and IL-1β precursor protein [41,42]. The elevated levels of cytokines IL-1β may induce a persistent inflammation, leading to the occurrence and development of pyroptosis [43]. In addition, a rencent study showed that melatonin could alleviate adipose tissue pyroptosis through inhibiting NF-κB/GSDMD signal in mice [29]. Moreover, the present study demonstrated that Rb2 obviously inhibited the phosphorylation levels of p65 and IκBα in the EAT of HFD-fed mice and 3T3-L1 adipocytes. To confirm it, we treated 3T3-L1 adipocytes with BAY, a specific inhibitor of NF-κB, and further observed that BAY treatment reduced the expression of the NLRP3 inflammasome and improved insulin sensitivity, indicating that the suppression of the NF-κB pathway contributed to the amelioration of IR by inhibiting pyroptosis.

In summary, the present study validated that Rb2 ameliorated IR via the inhibition of pyroptosis in 3T3-L1 adipocytes and EAT through the NF-κB pathway, providing a novel potential therapeutic approach to ameliorate IR in obesity.

## Supplementary Material

Supplemental Material
