# Supplementary figures and images for: Ginsenoside Rb2 improves insulin resistance by inhibiting adipocyte pyroptosis
Source: Adipocyte. 2020 Jun 24;9(1):302–12. doi: 10.1080/21623945.2020.1778826 (PMC7469678; doi:10.1080/21623945.2020.1778826)

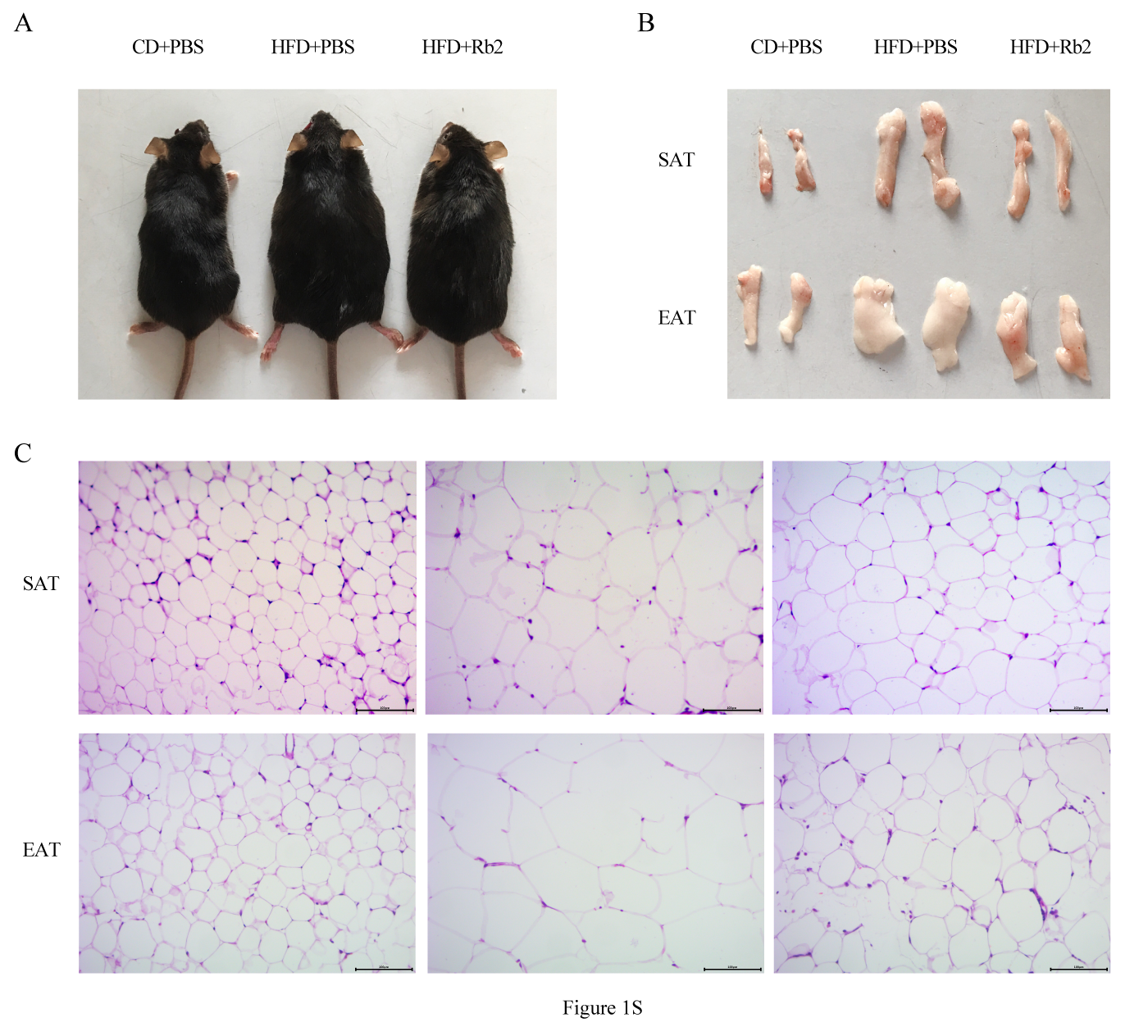

Supplement: Supplemental Material [file KADI_A_1778826_SM5454.tif]
